# 

**Published:** 1867-12

**Authors:** 


### CONTENTS FOR DECEMBER.

International Medical Congress,---------------------------------------- 561

Case Supposed to be Carcinoma of the Stomach. By M. A. McClelland, M.D., Knoxville, Ill.,---------------------------------------- 593

Case of Fibrous Tumor Removed from the Broad Ligament of Uterus. By Walter Burnham, M.D., Lowell, Mass. Reported by Chas. M. Clark, M.D., Chicago, Ill.,---------------------------------------- 596

Dr. Burnham's Report of the above Operation.------------------ 599

Shell Wound in the Forehead. By A. A. Dunn, M.D.,---------------- 601

Conservative Surgery. By C. F. Hart, M.D.,------------------------ 604

Chicago Medical Society,---------------------------------------- 606

Dangerous Effects of Chloroform. Chronic Asthma Relieved by Electricity. Rupture of the Spleen. Singular Fracture of the Os Calcis. Retained Placenta. Stricture, Enlarged Cancerous Liver. Death from large doses of Digitalis. Bending of the Radius and Ulna. Perforating Ulcer of the Ileum. Facial Paralysis in Syphilis. Tetrachloride of Carbon. Bromide of Potash in Epilepsy. Valvular Disease of the Heart,---------------------------------------- 606

CORRESPONDENCE.

Gelsemium. By D. L. Phares, M.D., Woodville, Minn.,---------------- 610

Letter from E. R. Hutchins, M.D., Philadelphia, Pa.,------------------ 612

Letter from J. B. Fares, M.D., Romeo, Mich.,------------------------ 614

Letter from H. O. Hitchcock, M.D., Kalamazoo, Mich.,---------------- 615

BOOKS RECEIVED.

Report on Meteorology, Medical Topography, and Epidemic Diseases of Illinois. By R. C. Hamill, M.D., Chicago, Ill.,---------------------- 612

Catalogue of the Surgical Section of the U.S. Army Medical Museum. Prepared under the direction of the Surgeon-General, U.S.A. By Alfred A. Woodhull, Assistant-Surgeon and Brevet-Major, U.S.A.,-- 616

A Contribution to the History of the Hip-Joint Operations Performed during the late Civil War. By Paul F. Eve, M.D., Prof. of Surgery in the University of Nashville, Tenn.,---------------------------- 616

The Gospel Among the Animals, or Christ with the Cattle. By Samuel Osgood, D.D.,---------------------------------------- 616

A Complete List of the Muscles of the Body. By W. Little, M.D., Chicago, 616

Lectures on the Diseases of Women. By Charles West, M.D., F.R.C.P.,-- 616

Seventeenth Anniversary Meeting of the Illinois State Medical Society,-- 617

EDITORIAL.

The December No. of the Journal for 1867,---------------------------- 618

Professorial Changes,---------------------------------------- 619

Nil Admirari,---------------------------------------- 620

A Voice from Lilliput,---------------------------------------- 621

Obituary,---------------------------------------- 622

Note from Prof. Rea,---------------------------------------- 622

Change of Base,---------------------------------------- 623

Receipts Acknowledged,---------------------------------------- 623

To the Medical Profession,---------------------------------------- 623

Winter Retreat for Consumptives,---------------------------------------- 623

